# Extreme Achalasia Presenting as Anorexia Nervosa

**DOI:** 10.1155/2012/985454

**Published:** 2012-10-04

**Authors:** P. J. Goldsmith, B. Decadt

**Affiliations:** Division of Surgery, Stepping Hill Hospital, Poplar Gove, Hazel Grove, Stockport SK2 7JE, UK

## Abstract

*Background*. Achalasia may lead to cachexia if not diagnosed in an early stage. Surgery in cachectic patients is hazardous and complications may result in a protracted recovery or even death. Different treatment options have been described. In this paper, we report a stepwise surgical laparoscopic approach which appears to be safe and effective. *Methods*. Over a one-year period, a patient with a body mass index (BMI) below 17 being treated for anorexia nervosa was referred with dysphagia. Because of the extreme cachexia, a laparoscopic feeding jejunostomy (LFJ) was fashioned to enable long-term home enteral feeding. The patient underwent a laparoscopic Heller myotomy (LHM) when the BMI was normal. *Results*. The patient recovered well following this stepwise approach. *Conclusion*. Patients with advanced achalasia usually present with extreme weight loss. In this small group of patients, a period of home enteral nutrition (HEN) via a laparoscopically placed feeding jejunostomy allows weight gain prior to safe definitive surgery.

## 1. Introduction

The incidence of eating disorders is becoming increasingly common in young women, whilst achalasia is a rare disorder of dysmotility of the oesophagus. In exceptional cases, these can overlap, but this is difficult to diagnose as patients with achalasia often avoid food and vomit [1, 2], identical symptoms to those with some eating disorders.

We present the case of a young girl treated for anorexia nervosa and admitted to a psychiatric unit for management. Definitive diagnosis and treatment was only made when the patient were referred for a feeding jejunostomy, and further investigations were performed.

## 2. Case Report

An 18-year-old female A-level student was admitted to a psychiatric department after being diagnosed with anorexia nervosa. She had uncontrollable vomiting after meals, loss of weight, and hypokalemia. Whilst she wanted to gain weight, she was unable to do so. Her vomiting had started four years earlier, and a gastroscopy, performed abroad, was found to be normal. The eating disorder was thought to be precipitated by feeling fat as a child and bullying by her mother for being overweight. Her mother and father had split up soon after the eating disorder was diagnosed.

Whilst at the psychiatric unit, her weight was 32.9 kg, height was 1.57 m, and body mass index (BMI) was 13.3. During the two months of admission in the psychiatric unit for assessment and treatment, there had been attempts to pass a nasogastric tube, but these had failed due to difficult placement. She developed a chest infection, which was treated by antibiotics, but the report of the chest X-ray raised the possibility of an oesophageal pouch. This was followed up by a barium swallow, which revealed a mega oesophagus, which tapered towards the gastro-oesophageal junction, consistent with a radiological diagnosis of established achalasia. 

She was subsequently transferred to an upper gastrointestinal surgeon for further investigations, and gastroscopy showed a narrowing at the gastro-oesophageal junction (GOJ), which was not possible to traverse. 

At this time, with a BMI of only 13.3, any further interventional treatment options, such as oesophageal dilatation or Heller myotomy, would carry a considerable morbidity/mortality in the event of any complications, due to her profound malnourished state. It was decided to place a feeding jejunostomy, inserted via a laparoscopic-assisted technique. Two months after feeding, a further gastroscopy was attempted, the stricture at the GOJ was negotiated, a balloon dilatation was performed, and biopsies were taken. Histology showed low-grade oesophagitis and no evidence of malignancy. It was our intention to perform manometry studies to confirm the diagnosis, but this failed due to inability to pass the manometry probe past the stricture. After 2 months of HEN, she was reviewed in clinic where weight gain had been sufficient and had risen to 46 kg (BMI 18.7). She was still vomiting after meal but was continuing with the jejunal feeding in the nighttime. At this point, it was felt safe enough to proceed to LHM. The patient made an uneventful recovery and was able to eat and drink normally without any vomiting. Feeding jejunostomy was removed, and the patient's weight stabilised to give a BMI of 22, and she went through delayed puberty.

## 3. Discussion

Anorexia nervosa (incidence 8 : 100,000 [3]) is a much more common diagnosis than achalasia (incidence 1.6 : 100,000 [4]), and there have been a number of case reports where the two diagnoses have been considered [5]. In the case, we reported that the patient had been suffering from these symptoms for four years before any formal treatment was initiated. A gastroscopy performed at the onset of symptoms was reported to be normal. This was either a misdiagnosis or the achalasia developed in the intervening period. The treatment of achalasia has classically been by pharmacological Botox injections used as a temporary measure, balloon dilatation, or surgical therapy in the form of a Heller myotomy. Heller myotomy performed laparoscopically is thought to be the most effective treatment with significantly less morbidity when compared to pneumatic dilatation [6]. All of the above options carry with them a risk of complications with the morbidity and mortality increased in a patient with severe malnutrition. LFJ has been used successfully in patients with obstructed upper gastrointestinal tract in cancer patients [7] but has never been described as a method of enteral feeding in achalasia prior to more definitive surgical management. It can be seen that the feeding over a three-month period was successful in raising the BMI form 13.3 to 18.7.

In the case, we have highlighted that there has been a considerable amount of time from the onset of the first symptom and the formalisation of definitive care for the patient. When presented with a patient with persistent vomiting and a desire to put on weight, the diagnosis should always be presumed to be due to a physical cause until thorough investigation proves otherwise. Achalasia should be considered in any patient presenting with difficulty in swallowing or dysphagia even when a diagnosis of anorexia nervosa is being considered. We would recommend investigations with barium swallow and gastroscopy and with oesophageal manometry if the findings suggest achalasia. If these investigations are found to be normal but the clinical picture does not fit with the diagnosis, these investigations should be repeated. It is also our opinion that some form of enteral feeding should be considered before a more definitive surgical procedure is performed, and our experience has shown LFJ to be a simple and effective way of achieving this.
